# A Summary of the SARS-CoV-2 Vaccines and Technologies Available or under Development

**DOI:** 10.3390/pathogens10070788

**Published:** 2021-06-22

**Authors:** Zainalabideen A. Abdulla, Sharaf M. Al-Bashir, Noor S. Al-Salih, Ala A. Aldamen, Mohammad Z. Abdulazeez

**Affiliations:** 1 Department of Clinical Sciences, Faculty of Medicine, Yarmouk University, Irbid 21163, Jordan; sharafalbasheer@yahoo.com; 2 Department of Basic Medical Sciences, Faculty of Medicine, Yarmouk University, Irbid 21163, Jordan; noor.saleh@yu.edu.jo (N.S.A.-S.); aldamin.alaa@yahoo.com (A.A.A.); 3 Internship Program, Princess Basma Teaching Hospital, Irbid 26125, Jordan; 2015899102@ses.yu.edu.jo

**Keywords:** vaccines, SARS-CoV-2, COVID-19, immune response

## Abstract

Since the beginning of 2020, the world has been in a race to develop vaccines that can control the COVID-19 pandemic. More than 250 projects have been initiated for this purpose, but only 14 of them have been authorized for use, despite being in phase 3 clinical trials. More than 40 other vaccines are also in phase 1/2 clinical trials and show promising outcomes. Regarding the appropriate choice of vaccines for each country or region, we reviewed the currently used vaccines in light of the different influencing parameters. These factors include the mode of action, dosage protocol, age group of the vaccinee, side effects, storage conditions, mounted immune response, and cost. Technically, there are seven types of vaccines developed against SARS-CoV-2: messenger RNA (mRNA), nonreplicating and replicating vectors, inactivated viruses, protein subunits, viral-like particles, DNA vaccines, and live attenuated vaccines. The mRNA type is being used for the first time in humans. Unfortunately, mutated variants of SARS-CoV-2 have started to appear worldwide, and researchers are investigating the effects of the currently used vaccines on them. There are many concerns regarding the long-term protection afforded by these vaccines and their side effects, and whether they require future modifications to be effective against the mutated variants. The development of new vaccines using more advanced technology is paramount for overcoming the difficulties in controlling the COVID-19 pandemic across the world.

## 1. Introduction

Amid the tsunami of the COVID-19 pandemic—with the first known cases reported on 31 December 2019—it was realized that vaccines could play an essential role in increasing the immunity of the population, preventing severe conditions caused by COVID-19 infection, reducing the burden on healthcare systems, and minimizing economic losses [1,2]. This crisis prompted an unprecedented race for the development of different vaccines using existing expertise in vaccinology [3]. Traditionally, vaccines require 10–15 years of research, development, and testing before their clinical usage can begin [4]. However, in early 2020, scientists embarked on attempts to produce safe and effective SARS-CoV-2 vaccines at record speed [5].

**Figure 1:**
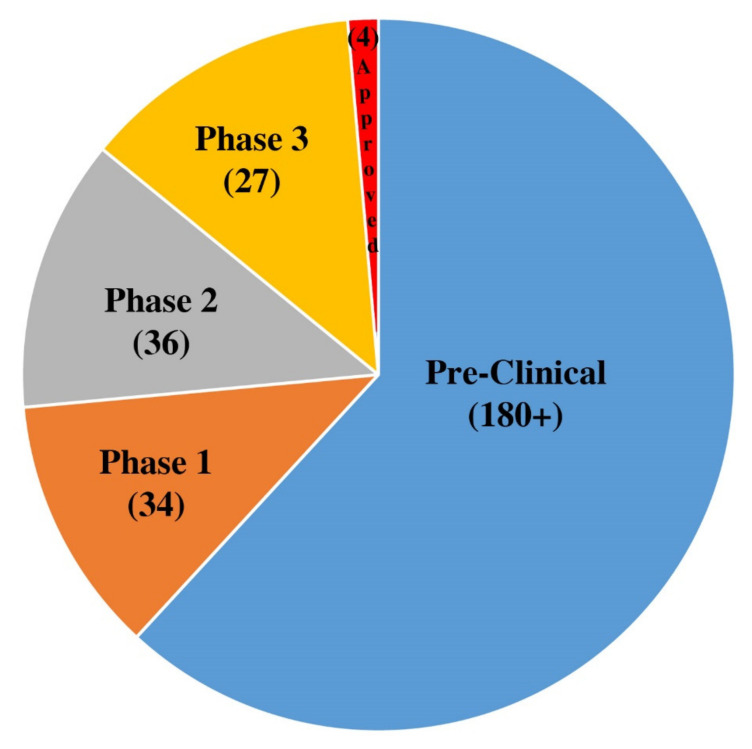
The different vaccines in preclinical phases, the three phases of clinical trials, and the authorized vaccines, based on WHO’s recently published numbers [7].

More than 250 vaccine projects were initiated worldwide in 2020, many of which involve conducting active preclinical trials in animals [6]. According to a recent WHO report, 97 vaccines are in clinical trials from phases 1 to 3, and 182 are in their preclinical development stages (Figure 1) [7,8]. Different technologies have been applied in vaccine preparation, some conventional and some newly developed and applied for the first time in humans [8]. Thus far, at least 14 vaccines have reached clinical application and/or have been authorized for use for use against SARS-CoV-2 (Table 1). 

**Figure 2:**
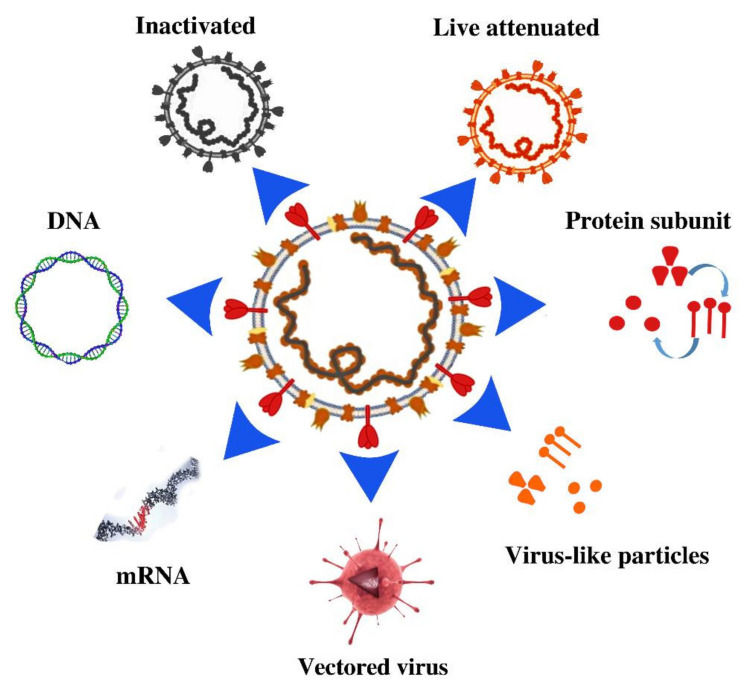
The different seven classes of vaccines against SARS-CoV-2 virus.

The vaccines against SARS-CoV-2 can be categorized into seven classes (Figure 2) [9]. The first comprises nucleic acid (RNA or DNA) vaccines; these consist of snippets of the virus’ genetic material, which are injected directly into human body cells. The second class comprises knocked-out virus vaccines, which use inactivated or weakened viruses. The third class is viral vector vaccines, which use Trojan horse nonreplicating vectors—or vectors that replicate much less frequently—to introduce a piece of transcribed DNA from SARS-CoV-2 to another unrelated virus, such as a modified adenovirus. The injected vectors instruct human cells to make coronavirus proteins and trigger an immune response. The fourth class comprises recombinant protein subunit vaccines, which use no genetic material but use whole or fragments of viral proteins packed into nanoparticles for better delivery and uptake by body cells. The fifth class is composed of coronavirus protein subunits; these can be synthesized and assembled to construct virus-like particles (VLPs) similar to those of natural SARS-CoV-2. The sixth class is DNA vaccines that are prepared from viral RNA by reverse transcription. The final class is a group of attenuated and repurposed vaccines based on already-established technology for vaccine preparation (Figure 2). Researchers are also evaluating more than 40 vaccines in phase 1/2 clinical trials on humans in various countries; the 43 vaccines that have most progress are listed in Table 2.

## 2. The Viral Spike (S) Protein

**Figure 3:**
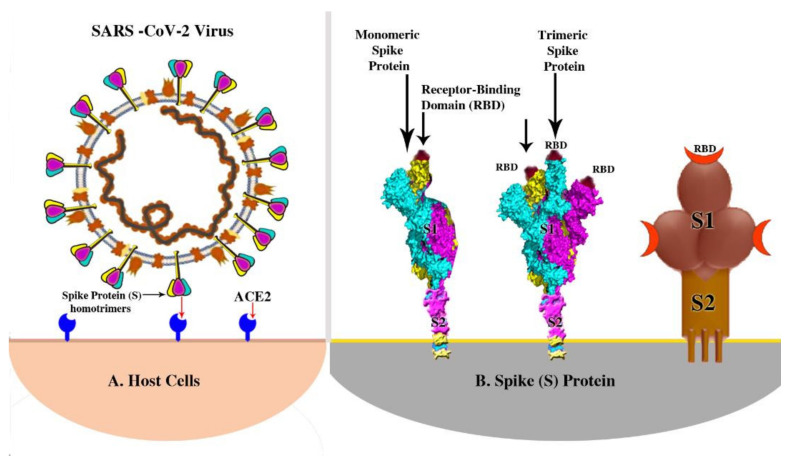
(**A**) SARS-CoV-2 interacts with high affinity with the angiotensin-converting enzyme-2 (ACE2) receptor. (**B**) The spike protein consists of two domains, S1 and S2 in both monomeric and trimeric types. Receptor-binding domain (RBD) is shown in the three spike protein pictures.

The S protein is frequently considered the major antigen target for vaccines against the SARS-CoV-2 virus. The genome of SARS-CoV-2 encodes one large spike protein (S) that plays a pivotal role during viral attachment and entry into host cells. The spike protein can be cleaved into S1 and S2 subunits by host proteases, each of which has a trimeric structure [6]. The S1 domain is distally located and contains the receptor-binding domain (RBD) that binds with high affinity to the cellular receptor ACE2. S2 is the proximal domain, and its cleavage by proteases on the host cell surface alters its conformation and enables the viral envelope to fuse with the cell membrane of an infected cell (Figure 3). Neutralizing antibodies target both the S1 and S2 domains. Antibodies against the S1 RBD block the interaction with ACE2 receptors, while those against S2 can block membrane fusion (see Section 7). The S protein exists in a so-called metastable prefusion conformation, and proteolytic cleavage refolds the protein into a more stable, postfusion state.

## 3. Vaccines Approved for Use

There are fourteen specific vaccines of different categories that have been given authorization for use for combating COVID-19 (Table 1), which are discussed below in their respective categories.

### 3.1. Messenger-RNA (mRNA) Vaccines

mRNA vaccines are characterized by robust immunogenicity, intrinsic adjuvant properties, low costs for preparation and production, favorable safety profiles, quick production, and special storage and delivery systems [72]. 

This is a sophisticated vaccine preparation technology that is being used in humans for the first time. mRNA vaccine preparation has been investigated over the last 20 years for different viruses, such as rabies, influenza, and Zika [73]. However, mRNA vaccines for COVID-19 are the first to be approved and used in humans. The main advantage of this technology is that it allows the body’s cells to produce S proteins rather than injecting them as in vaccines. This reduces the time required for building the vaccine and hence requires less time compared to that required for classical vaccines [74]. mRNA vaccines utilize manufactured nucleoside-modified, single-stranded messenger RNA (mRNA) to deliver genetic instructions to human cells for building up the coronavirus protein known as the spike protein (S). The mRNA enters the human cells encapsulated by lipid nanoparticles (LNP) that prevent the cells of the body from degrading it and give stability to the mRNA, which is a fragile molecule. The mRNA does not linger in the body’s cells for more than 48 h. Once it has passed its instructions to the protein-making machinery in the cytoplasm of the body’s cells, enzymes called ribonucleases (RNases) degrade the mRNA [75]. Therefore, it is impossible for the mRNA to move into the nuclei of cells, as it lacks the genetic signal that would allow it to enter this forbidden compartment; thus, the RNA cannot integrate with the DNA of the vaccinated cells, posing no risk of inducing genetic changes. After the S protein is produced by the cells of the body, the immune response is initiated with its two arms, i.e., humoral (antibodies) and the T-cell (CD4+ and CD8+ T cells)-mediated immunity. The neutralizing antibodies can stop the spike protein or its floating fragments from attaching to cells. The killer T cells (CD8+) in vaccinated individuals recognize and destroy any coronavirus-infected cells that display the spike protein fragments on their surfaces. Three important mRNA vaccines currently in use in humans that are authorized for use are given in the following subsections.

#### 3.1.1. Pfizer–BioNTech Vaccine (PBV)

The PBV vaccine is produced by Pfizer, an American multinational pharmaceutical corporation based in New York, in association with the German company BioNTech. The generic name of this vaccine is tozinameran (Comirnaty) [10,11,76]. The PBV is given in two doses 3 weeks apart [77]. It is also recommended that vaccinated individuals receive a booster shot, or a third dose, within 12 months of being fully vaccinated and then annually thereafter [76,77,78]. It is supplied in vials containing enough liquid for five shots, although it may be adequate for two extra shots. According to the data released by the manufacturers, this vaccine is 95% efficacious in protection [77]. Its efficacy in elderly people is almost the same (94%) as that in those under 65 years of age [78]. It offers strong protection against COVID-19 within 10 to 14 days of the first dose regardless of the recipient’s race, weight, or age [79]. It can produce strong antibody and T-cell immune responses. This vaccine does not cause any serious side effects but frequently causes short-lived symptoms such as pain at the site of injection, mild fever, fatigue, and muscle pain [80]. The PBV requires an ultra-cold temperature of −70 °C (−94 °F) for storage and distribution, which imposes difficulties on its usage in certain countries. However, recent reports claim that it can be stored at a much higher temperature (−20 °C) for 2 weeks [81]. Mr. William Shakespeare (81-year-old) was the first man to be vaccinated with the Comirnaty vaccine on 8 December 2020 [82]. The recommended age group for vaccination is >16 years, although other age groups are also currently under investigation [83]. The FDA has recently lowered the age at which people can receive Pfizer’s COVID-19 vaccine in the United States to include children aged 12–15 years. This move is expected to result in millions more shots being administered [83].

#### 3.1.2. Moderna Vaccine (MV)

The MV is manufactured by a Massachusetts-based company, Moderna, in collaboration with the U.S. National Institute of Health. This is an mRNA-based vaccine (mRNA-1273) encapsulated in LNP [12]. This vaccine has an advantage over PBV, in that it can be stored at temperatures equivalent to a standard freezer (−20 °C), making it easier to ship to remote and rural areas [84]. It has an efficacy of 94.1% according to the data of the manufacturers and the U.S. FDA [85]. The MV is used for people over 18 years of age [85]. It requires two shots four weeks apart and does not raise any safety concerns. There are clinical trials to investigate the usage of MV for children, for people with cancer and for those in pregnancy. Preliminary reports showed that children and adolescents (12–17 years) exhibited stronger immune responses to the vaccine and consequently showed more intense side effects such as fever and aches [86,87]. However, after the administration of millions of doses globally, the Moderna vaccine does not show an association with cerebral venous sinus thrombosis (CVST) or thrombotic events, based on analyses of recent data [86,87].

#### 3.1.3. CVnCoV Vaccine of CureVac (CVV)

The CVV is produced by Tübingen’s CureVac biotech firm in partnership with the giant pharmaceutical company Bayer and is currently in its combined phase 2b/3 clinical trial. This vaccine is considered a rival to the leading mRNA vaccines of Pfizer–BioNTech and Moderna [13,88]. The CVV utilizes a natural, nonchemically modified, synthetic mRNA coding the prefusion-stabilized full-length spike protein of SARS-CoV-2. Therefore, CVnCoV is unlike the Pfizer–BioNTech and Moderna COVID-19 vaccines, which use nucleoside-modified RNA [13,88]. The CVV is administered via a two-dose regimen, with the doses administered four weeks apart intramuscularly. This vaccine requires lower doses (12 micrograms) than the 30 micrograms for PBV and the 100 micrograms for MV [88,89]. The manufacturers claimed that it showed an efficacy of 95% (comparable to that of PBV and MV). Furthermore, it can be stored at 5 °C (41 °F) and is stable for three months at refrigerator temperatures of 2–8 °C (36–46 °F), which makes it suitable for usage in poorer countries [90]. This is a further advantage over the two above-mentioned mRNA vaccines.

### 3.2. Human Adenovirus Nonreplicating and Replicating Vector-Based Vaccines

These types of vaccines utilize replication-deficient viral vectors (backbone) or attenuated replication-competent (bioengineered) viral backbones [44,91,92,93]. The most common replication-incompetent or defective viral vectors currently in use are adenoviruses for carrying and delivering a selected plasmid-manufactured, double-stranded DNA segment of the RNA of SARS-CoV-2 that codes the S-protein antigen of the virus. The adenovirus vectors currently in use by different manufacturers are human Ad5 and Ad26 adenoviruses and a modified version of the chimpanzee adenovirus ChAdOx1 [92].

After injection, the vectors then enter the body’s cells but cannot replicate intracellularly. The delivered genetic material escapes from the vectors and travels to the nucleus, where the DNA is stored but does not integrate with the body’s DNA [44,91,92,93]. Afterwards, it is transcribed into mRNA that leaves the nucleus to be read and “translated” into spike proteins; these proteins begin to be assembled on the surfaces of infected cells. Once the S proteins or their fragments are recognized by the immune system, it starts to send warning signals and generate specific neutralizing antibodies and activated T cells (CD4+ and CD8+), as well as memory cells of the B- and T-cell types. The protection generated from these vaccines ranges between 62 and 90% (average at 70%) [19,94]. The vectors used with these vaccines have a tough protein coat that helps in protecting the genetic material inside them. For this reason, the vaccine does not have to stay frozen and can be stored for at least 6 months at refrigerator temperatures (2–8 °C) [95]. Moreover, these vaccines can induce strong immune responses and do not require adjuvants to be incorporated with them. This is because these vaccines contain their own pathogen-associated molecular patterns (PAMPs) that can serve as built-in adjuvants [44,91,92,93].

Furthermore, weakened but replicating viral vectors such as measles, influenza, and vesicular stomatitis virus (VSV-MERK) can also be used to carry genes for the spike protein of SARS-CoV-2 [19,44,91,92,93,94]. In addition, modified vaccinia virus Ankara (MVA-MERS), which is an attenuated orthopoxvirus infecting avian cells but not mammalian ones, can also be used as a vector for the preparation of vaccines with a predilection for protecting mucosal surfaces, such as those of the respiratory tract [96,97].

Four adenoviral, nonreplicating, vector-based vaccines (Table 1) are currently under authorized for use in humans, and they are given in the following subsections.

#### 3.2.1. Oxford–AstraZeneca Vaccine (OAV; AZD 1222; Vaxzevria)

The OAV is produced by Oxford University in cooperation with the British–Swedish company AstraZeneca, along with its Indian version, which is called Covishield [98]. The OAV is a vector vaccine that utilizes a vector based on the recombinant ChAdOx1 of chimpanzees. It is given to individuals >18 years of age in two doses of 5 × 10^10^ viral particles each (standard doses, “SD/SD”). Moreover, the developers tried a half dose as the first dose (low dose, “LD”) and a standard dose as the second dose in a regimen described as “LD/SD” [99]. The AZD 1222 vaccine has an acceptable safety profile and is efficacious in combating symptomatic COVID-19. In addition, this vaccine is effective against the new and more contagious U.K. SARS-CoV-2 variant B.1.1.7, and partially (10% efficacy) against the South African B.1.351 variant (see below) [100].

The OAV was reported to have an efficacy ranging from 62 to 90%, according to the two-dosage protocol of SD/SD or LD/SD, respectively [14,99]. Furthermore, this vaccine can be kept at refrigerator temperatures, 2–8 °C, for at least six months, which makes it easy to store, transport, and distribute globally [14,99]. The OAV triggers strong humoral and cellular immune responses. Again, this vaccine produces minor side effects, such as fatigue and headache [99].

It has been recently reported that individuals diagnosed with thrombocytopenia syndrome (TTS) within 3 weeks of vaccination with Vaxzevria should be actively investigated for signs of thrombosis. In addition, individuals who present with thrombosis within 3 weeks of vaccination should be evaluated for thrombocytopenia [101]. A clear contraindication is issued in some countries to not vaccinate such individuals with Vaxzevria [100,101]. Further analysis also found another link with a condition called heparin-induced thrombocytopenia (HIT) in people taking the anticoagulant heparin. HIT is thought to be triggered when heparin binds to a protein called platelet factor 4. This stimulates an immune response via the production of antibodies against platelet factor 4 that results in platelet destruction and the release of clot-promoting material. The presence of leg pain, seizures, and a change in mental status are considered possible signs and symptoms of TTS. Vaccinated individuals with severe or persistent headaches, blurred vision, skin bruising beyond the site of vaccination after a few days, shortness of breath, chest pain, leg swelling, or persistent abdominal pain are advised to consult experienced health professionals or specialists in hematology and/or in coagulation to investigate, diagnose, and treat possible TTS, as this condition requires urgent management. Mild thrombocytopenia is commonly reported in fewer than 1 in 10 vaccinated persons. The risk of TTS is lower after the second dose, with an estimated rate of 1.7 cases per million doses. Finally, Guillain–Barre Syndrome (GBS), a rare autoimmune disease, is under investigation to explore any link with the Vaxzevria vaccine’s side effects [101].

#### 3.2.2. Sputnik-V Vaccine (SVV)

The SVV vaccine was named in memory of the Soviet-era satellite program [102]. This Russian vector-based vaccine is produced by the state Research Centre of Virology and Biotechnology, Gamaleya Institute. It utilizes a combination of two adenoviruses (Ad5 and Ad26) that are not recognized by the human immune system as foreign and, hence, are not destroyed [15,103]. The developers stated that it had an efficacy of 91.6% in protection after two doses administered three weeks apart intramuscularly [103]. In the first dose, the Ad26 vector was used, while in the second, the Ad5 vector was utilized. However, Russian scientists tested a one-dose version (Sputnik-light), which could provide temporary immunity for 3 to 4 months and has a claimed efficacy of 73–85%; this could help countries with high infection rates [104]. It can be stored at a standard freezer temperature of −20 °C [105]. Recently, it was announced that AstraZeneca and Sputnik-V developers are testing a combination of both vaccines to see whether it could improve their efficacy [106]. Reports from Russia’s Gamaleya Institute denied a link between the Sputnik-V vaccine and the formation of blood clots [15,103].

#### 3.2.3. Johnson and Johnson Vaccine (J&J V; JNJ-78436735)

The JJV is manufactured by Janssen Pharmaceutical, which is owned by Johnson and Johnson Multinational Corporation. The JJV utilizes the Ad26 adenoviral vector, which was used for the Ebola vaccine by the same company. The company applied for an Emergency Use Authorization (EUA) from the U.S. FDA in February 2021 [16,107]. It is a one-dose vaccine that can produce a neutralizing antibody response in 90% of vaccinated people after four weeks and in all recipients after two months. In a phase 3 clinical trial (ENSEMBLE), the manufacturers also investigated a 2-dose regimen to see whether it can confer longer protection and increased antibody levels [108]. The JJV shows an efficacy of 66% globally and 72% in the United States [107]. It can be stored for up to 3 months at refrigerator temperatures (2–8 °C; 36–46 °F) and for two years at −20 °C (−4 °F) [109]. It showed a 66% effectiveness in preventing infection after a single dose and was capable of preventing 85% of severe COVID-19 cases 28 days after vaccination [110]. It is also capable of protecting against the SARS-CoV-2 variant of the B.I.351 lineage observed in South Africa [107]. The vaccine is well tolerated and shows no serious side effects [111]. However, there are also reports linking clot formation with the JJV. The blood clots that have been tentatively linked to the AstraZeneca and J&J vaccines have particular characteristics: they occur in unusual parts of the body, such as the brain or abdomen, and are coupled with low levels of platelets, cell fragments that aid blood coagulation [111].

#### 3.2.4. AD5-nCoV (Convidecia) Vaccine

The AD5-nCoV vaccine is prepared by the Chinese CanSino Biologics Company in cooperation with the Academy of Military Medical Sciences. The Convidecia vaccine is based on using the Ad5 adenovirus vector, as reflected in its official name [17,42]. It is currently in phase 3 clinical trials, and the Chinese government has already approved it for military use, for a period of one year. Furthermore, the vaccine has also been awarded authorization for use in some countries. The efficacy of the vaccine after a one-shot dosage is 65.7% (comparable to that of the Johnson and Johnson JJV vaccine; see above) [17,112,113]. It has the advantage of being suitable for storage at refrigerator temperatures (2–8 °C). No serious adverse reactions after vaccination have been reported.

### 3.3. Inactivated Coronavirus Vaccines

Researchers picked up one of three viral variants that can multiply in monkey kidney cells and grow in bioreactor tanks. Dousing large stocks of this variant with a chemical called beta-propiolactone could disable the viruses by binding to their genes and thus preventing their replication; however, their proteins, including the spike (S) protein, remained intact [114]. The preparation was then mixed with an aluminum-based adjuvant to boost the immune response against the inactivated vaccine [115].

Four inactivated vaccines have been given authorization for use (Table 1). Other inactivated vaccines are in phase I/II clinical trials or preclinical trials (Table 2). The inactivated vaccines express a wide range of native viral antigens [18,19,20,21]. Such multiple antigens can induce a TH2 response and lung eosinophilia, which may be worse in aged hosts [116]. This broad-spectrum immune stimulation may result in a special condition in the postvaccination period called the vaccine-related enhancement of disease (VRED) [117]. This condition may also be triggered by the aluminum adjuvant used in the vaccine, which is also known to drive TH2-cell immune responses (see Section 7). Therefore, TH1-skewing modified alum or other types of adjuvants such as CpG are recommended as alternatives to avoid VRED [118].

It is possible for an inactivated virus vaccine to induce a broader immune system response than vaccines that only feature the spike protein. Since the existing viral variants of concern (see Section 5) have critical mutations in the spike, inactivated virus vaccines could theoretically offer an advantage for protection against these variants [18,19,20,21,118].

#### 3.3.1. Sinopharm Vaccine (SV; BBIBP-CorV)

The SV vaccine is manufactured by Sinopharm Group, which is a state-owned Chinese company, and is marketed with the cooperation of the UAE. It is an inactivated vaccine and is administered in a two-dose regimen, with the doses given 3 weeks apart by intramuscular injection. It showed an efficacy of 79.34% in China and 86% in the UAE, besides being 100% effective in preventing moderate and severe COVID-19 cases [18,119]. The developers did not report any serious side effects during its phase III clinical trial or after its authorization for use [120].

The available data on the Sinopharm vaccine in pregnant women are inadequate in informing us on either the vaccine’s efficacy or vaccine-associated risks during pregnancy. However, this is an inactivated vaccine with an adjuvant that is commonly used in many other vaccines with proven good safety profiles, including in pregnant women. The effectiveness of the Sinopharm vaccine in pregnant women is, therefore, expected to be comparable to that observed in nonpregnant women of similar ages [18,119,120].

#### 3.3.2. Sinopharm-Wuhan Vaccine (SWV)

The SWV vaccine was prepared by the Chinese Wuhan Institute of Biological Products. It is Wuhan’s version of the Sinopharm vaccine. It is effective in preventing COVID-19 in 72.5% of vaccinees [121]. It shows comparable side effects to the Sinopharm vaccine and is also in its phase III clinical trial [19].

The Wuhan vaccine utilizes the WIV-04 strain, which was isolated and cultivated in a Vero cell line for propagation. Then, the supernatant of the infected cells was inactivated as described above. Interim analysis of two randomized controlled trials showed a seroconversion rate of 100% in the phase 1 trial and 85.7% in the phase 2 trial [19,121]. This vaccine is given in two doses 3–4 weeks apart. A third dose is recommended for those individuals who show weak immune responses. A lower-dosage injection was associated with a higher geometric mean titer (GMT) of neutralizing antibody at Day 14 after the third injection, compared with the other dosage protocols [19,121].

#### 3.3.3. CoronaVac Vaccine (CV; Formerly PiCoVacc)

The CoronaVac vaccine is manufactured by a private Beijing-based biopharmaceutical company, SinoVac Biotech, in collaboration with the Brazilian research center, Butantan. The CV vaccine is given in two doses 2 weeks apart by intramuscular injection [20]. The Chinese manufacturing company reported 50.38% efficacy in the Brazilian trial when including “very light cases” in their data analysis [122]. Furthermore, the Brazilian trial showed efficacy of 78 and 100% in preventing mild and severe COVID-19 cases, respectively [122,123]. The interim analysis of other countries’ clinical trials showed higher efficacy of 83.5 and 65.3% in Turkey [124] and Indonesia [125], respectively. Moreover, it was reported that this vaccine generates a moderate immune response with lower antibody levels in comparison with levels in patients who have recovered from COVID-19 [126]. Therefore, this vaccine requires an adjuvant, such as alum, to boost the immune response, but this requirement in turn makes the vaccine unsuitable for respiratory administration [19,126]. The safety and effectiveness in children 3–17 years of age is also being studied in clinical trials, with promising results [127]. CoronaVac showed no serious side effects. It can be stored at refrigerator temperature (2–8 °C; 36–46 °F), making it suitable for worldwide distribution [123].

#### 3.3.4. Covaxin Vaccine (COV; Bharat Biotech Vaccine, BBV152)

Covaxin vaccine was manufactured by the Indian Bharat Biotechnology Company in collaboration with the Indian Council of Medical Research and National Institute of Virology [21]. The COV has been granted permission in India for restricted use in emergency situations despite being in phase 3 of clinical trials [128,129]. The Indian company also signed a partnership with the Pennsylvania-based company Ocugen for marketing the vaccine in the United States [130]. This vaccine is used in a two-dose regimen with the doses given 4 weeks apart, and its efficacy is reported to be 81% [128,130], although 82.8 to 91.9% of the vaccinated people generated antibodies (seroconverted) with robust immune responses [131]. It can be stored for one week at room temperature, which makes it suitable for usage in tropical and subtropical countries [128].

### 3.4. Recombinant Protein Subunit Vaccines

These types of vaccines utilize no genetic materials but use whole or fragments of viral proteins packed in nanoparticles [49,132,133]. This type of vaccine is considered very safe and incapable of causing disease. Five vaccines of this type are in preclinical trials utilizing different protein (peptide) subunits [134]. Since these subunits are poorly immunogenic, they require adjuvants and repeated administrations [49,133]. They can primarily induce reasonable CD4+ T-cell activation and specific neutralizing-antibody responses, but they show poorer stimulation of CD8+ T cells [135]. Three types of recombinant protein subunit vaccines are described in the subsections below; they are in the late stages of phase 3 clinical trials or have received authorization in some countries.

#### 3.4.1. Novavax (NVX-CoV2373) Vaccine

The NVX vaccine is manufactured by a Maryland-based company, Novavax, in collaboration with GSK and Sanofi, two companies in the United Kingdom and France, respectively, by attaching viral proteins onto a nanoparticle carrier (microscopic particle) to aid efficient delivery and uptake by body cells [22]. It is administered in two doses three weeks apart by intramuscular injection. It can produce a strong antibody response (better than in COVID-19-recovered patients), as well as T-cell activation [136,137]. It is stable at refrigerator temperatures and has an efficacy of 89.3%, reaching up to 96% in a U.K. clinical trial [135,136,137]. Furthermore, this vaccine is under further investigation to see whether it can be given together with the flu vaccine [138].

#### 3.4.2. EpiVacCorona Vaccine (EVCV)

The EVCV vaccine is manufactured by the Vector Institute, a Russian biological research center. It is based on using fragments of synthetic viral peptides reflecting SARS-CoV-2 antigens [23,139]. It is given in two doses three weeks apart by intramuscular injection to people over 18 years of age as well as older people >60 years of age [139,140]. The developers claimed that it is stable during storage at refrigerator temperatures for up to two years. Its efficacy is officially unknown, and it is awaiting regulatory approval. However, all the volunteers who were administered the EVCV developed specific antibodies against its antigens [140].

#### 3.4.3. ZF 2001 (RBD Dimer) Vaccine

The developers of this vaccine are the Chinese Anhui Zhifei Longcom and the Academy of Military Medical Sciences. The vaccine uses a section of the spike protein named the receptor-binding domain (RBD) combined with an adjuvant, so it is considered very safe [24,133,141]. The ZF 2001 vaccine is administered in a three-dose course with the doses given 4 weeks apart by intramuscular injection [142]. The efficacy of this vaccine is officially unknown, as it is in a phase 3 clinical trial, but it has been approved for emergency use in Uzbekistan and China [143,144].

### 3.5. Virus-Like Particle (VLP) Vaccines

The VLPs are composed of several structural viral proteins (co-expressed or admixed) [145,146]. These VLPs are manufactured viral proteins—S, M, and E with or without N—co-expressed and budding from eukaryotic producer cells [147,148]. These particles are similar to the virus but lack the viral genome. Of these VLPs, the S can bind and enter via the ACE2 receptor. Additionally, the S particle can crosslink B-cell surface receptors and stimulate antibody production. These vaccines require adjuvants and repeated administration. Therefore, they are based on a noninfectious virus-like-particle (VLP) that resembles the morphology and structure of the SARS-CoV-2 particles but does not contain any of its infective genetic materials, thus rendering it extremely safe to produce and handle [146,147,148]. These VLPs can be produced in any biosafety level 1 facility. None of the VLP vaccines have yet been approved for use, but there are three promising VLP vaccines under development. Firstly, the Canadian company Medicago has genetically engineered plants to produce a VLP vaccine, which is in phase 2/3 clinical trials and was recently granted Fast Track designation by the U.S. FDA [149]. Secondly, the ContiVir team at the Max Planck Institute for Dynamics of Complex Technical Systems (Magdeburg, Germany) has designed and produced a virus-like particle vaccine [145,150]. Thirdly, a Georgia-based biotechnology company, GeoVax Atlanta, has used MVA viral vectors to express VLPs [151]. The last two vaccines are in preclinical trials.

### 3.6. Repurposed and Live Attenuated Vaccines

The Bacillus Calmette–Guerin (BCG) vaccine was prepared as a bacterial live attenuated vaccine at the beginning of the twentieth century to prevent tuberculosis [24]. Recently, the Murdoch Children’s Research Institute, Australia, in collaboration with the University of Melbourne, investigated whether BCG could partly protect against SARS-CoV-2 (repurposed) in a trial called BRACE. Evidence is not yet available, and recommendations from the WHO are awaited [152].

Furthermore, it is worth mentioning that there are three live attenuated vaccines for SARS-CoV-2 currently in preclinical trials in India and Turkey [6]. The COVI-VAC vaccine, developed by the Serum Institute of India, India, in collaboration with Codagenix, a New York private biotech company, is an example of a live attenuated vaccine under development. This technology is expected to lead to the development of a vaccine that can recognize the whole virus and be administered via the intranasal route, which would be a great advantage [125].

## 4. Vaccines in Phase 1/2 Clinical Trials

The most important vaccines currently in phase 1/2 clinical trials according to WHO press releases comprise 56 vaccines against SARS-CoV-2 (Figure 1) [135]. The current review has selected 43 leading vaccines from the seven different classes of COVID-19 vaccines mentioned above (Figure 2, Table 2). The categories and the number of candidate vaccines in each include mRNA (5 vaccines), DNA (8), vectors (10), protein subunits/viral-like particles (17), inactivated viruses (5), and live attenuated (1) vaccines. These vaccines are promising according to preliminary results, and some utilize modern technology in vaccinology for the production of vaccines that can be administered via oral [32,40], nasal [41,42,71], or dermatological [29,30,34,35,36] routes. Other vaccines also use vectors different than those vaccines currently awarded authorization for emergency use status or various purified VLPs (Table 2). Many more vaccines are in preclinical trials or animal studies; they are expected to be developed using advanced and sophisticated technology for their preparation (Figure 1).

## 5. Mutations and Types of Viral Variants

The world is currently facing mutated variants of SARS-CoV-2, which are further increasing the infection rate and threatening the effectiveness of the already prepared vaccines. The importance of these variants arises from their potential for increased transmissibility, increased virulence, or resistance to the vaccines available to protect individuals against them. Furthermore, they show higher mortality and morbidity rates, less susceptibility to antiviral therapy, capabilities to evade the usual diagnostic tests and natural immunity, ability to infect vaccinated individuals, and greater capabilities to infect immunocompromised patients.

There are thousands of different variants of SARS-CoV-2 circulating across the world. However, researchers around the world have identified seven notable variants of clinical significance: B.1.1.7 in the United Kingdom [153], B.1.429 and B.1.427 in the United States [154], B.1.525 in the United Kingdom and Nigeria [155], B.1.251 in Nigeria [156], B.1.351 in South Africa [153], B.1.617 (V1, V2, and V3) in India [157], and P.1 in Japan and Brazil [158]. B.1.1.7 is more transmissible (30–50%) globally, more lethal, and more virulent, but of unchanged antigenicity compared to the “sequence zero” original virus [153]. B.1.429 and B.1.427 have higher transmissibility and decreased sensitivity to neutralizing antibodies [154]. B.1.525 and B.1. 251 both show a moderate reduction in their ability to be neutralized by antibodies [155,156]. The B.1.351 mutation makes the virus bind more effectively to the ACE2 receptor on human cells, which facilitates its transmission, reduces its antigenicity, and makes neutralization by antibodies significantly less effective. The three Indian variants (B.1.617: V1, V2, and V3) but particularly V2 have significantly higher transmissibility (160%) than the original viral strain but slightly reduced susceptibility to antibody neutralization [157]. Lastly, the P.1 mutation increases viral transmission and lethality and reduces its susceptibility to antibody neutralization [158].

Although the WHO is trying to introduce uniform nomenclature for all of the viral variants, there are three main clades—GISAID (2021), Nextstrain (2017), and Pangolin (2020)—used to categorize these variants. Moreover, it has been found that the genetic codes of these variants are slightly different from each other and from the original virus. A mutation at N501Y is seen in the U.K., South African, and Brazilian variants. A mutation at E484K is seen in the South African, Brazilian, and some U.K. variants. Lastly, mutations at P681R and L452R might help the Indian variant to spread rapidly.

Scientists are now investigating the efficacy of the different vaccines against these variants and are taking the necessary measures to modify the current vaccines to make them effective against these variants. It is worth mentioning that SARS-CoV-2 has not mutated enough to render the current vaccines ineffective [157]. Future monitoring for the development of new variants of SARS-CoV-2 is therefore required. The current vaccines were designed for earlier versions of the coronavirus; there is evidence that they should work against the new versions but potentially less effectively. The latest research suggests that two doses of either the Pfizer or AstraZeneca vaccine are still protective against the Indian variant and that Pfizer is still protective against the new variants mentioned above. The AstraZeneca vaccine protects against the U.K. variant but is less effective against the South African strain, while the Moderna vaccine is effective against it but with weaker and a shorter-lived immunity. Lastly, the Moderna vaccine is still effective against the U.K. and N501Y variants [155,156,157,158].

## 6. Side Effects, Precautions, and Contraindications

No serious side effects have been reported from the usage of the currently authorized vaccines [159]. However, mild to moderate postvaccination symptoms have been reported, such as pain, swelling, and erythema at the local injection site; fever; chills; fatigue; myalgia; arthralgia; and axillary lymphadenopathy. One local symptom and systemic symptoms occur in 80–90% and 55–85% of vaccinated individuals, respectively [80,125,159]. These symptoms could be more pronounced after the second dose. All of these symptoms could be alleviated by the usage of acetaminophen, and most of them subside within 2 to 3 days.

Although very rare, cerebral venous sinus thrombosis has been reported with the Oxford–AstraZeneca vaccine [160], as well as an unexplained illness with the Johnson and Johnson vaccine [161], and gastrointestinal manifestations of nausea, vomiting, and diarrhea have been observed with the CanSinoBIO vaccine [41]. These clinical manifestations and a few others could be coincidental and not vaccine-related [41,160,161].

The side effects during pregnancy are similar to those that occur in nonpregnant people, and there is no need for women to avoid pregnancy if vaccinated. In addition, lactating women need not avoid vaccination [162]. It has been reported that patients with severe hypersensitivity, such as anaphylaxis, should avoid mRNA vaccines for the time being, but that avoidance is not indicated for local urticaria, mild allergic reactions, or latex allergy. Hypersensitivity was reported in 0.63 and 1.5% of people vaccinated with the Pfizer–BioNTech and Moderna vaccines, respectively [80,85,163]. Any history of anaphylaxis in response to other vaccines or injectable therapies should be assessed carefully by specialists to determine whether it represents an absolute contraindication. Anyone experiencing anaphylaxis after the first dose of a SARS-CoV-2 vaccine should not receive a second dose. It is worth mentioning that the vaccines’ vial stoppers do not contain natural rubber latex, so latex allergy is not a contraindication. Allergic individuals (reactogenic) should wait for at least half an hour after vaccination at the injection facility to ensure stable conditions. Other individuals are only asked to wait for 15 min after vaccination [85,163].

An immunocompromised status is not an absolute contraindication, as the risk of COVID-19 in immunosuppressed patients outweighs any risk from vaccination, so such patients should be vaccinated. Moreover, patients with autoimmune diseases, such as Guillain–Barre syndrome can be vaccinated if there is no contraindication [164]. Finally, although cases of Bell’s palsy has been reported in the postvaccination period in trials, these were later considered coincidental, and individuals who had Bell’s palsy can take the vaccine if desired. They should be handled in a similar way to others.

For patients with COVID-19 infection or their contacts, vaccines can be deferred until the end of the quarantine period of isolation to avoid exposing healthcare workers or other persons to the risk of infection. It is preferable to delay the vaccination of these individuals until after 90 days, since reinfection is uncommon at this point [164]. Vaccination should be offered regardless of prior history of asymptomatic or symptomatic SARS-CoV-2 infection. Serological tests for COVID-19 are not recommended for the purpose of vaccination. A fully vaccinated individual would achieve their maximum immune response two weeks after the last dose [165]. Persons who have received monoclonal or polyclonal antibodies (convalescent plasma) can be deferred from vaccination for 90 days as a precautionary measure [164]. Finally, there is limited information on the effect of vaccination in reducing transmission and how long it lasts. Thus, vaccinated individuals should continue to follow all the current guidance to protect themselves by wearing masks, maintaining 6-foot distancing, washing hands, abiding by travel regulations, and avoiding gathered crowds [166,167].

## 7. The Immune Response in the Postvaccination Period

**Figure 4:**
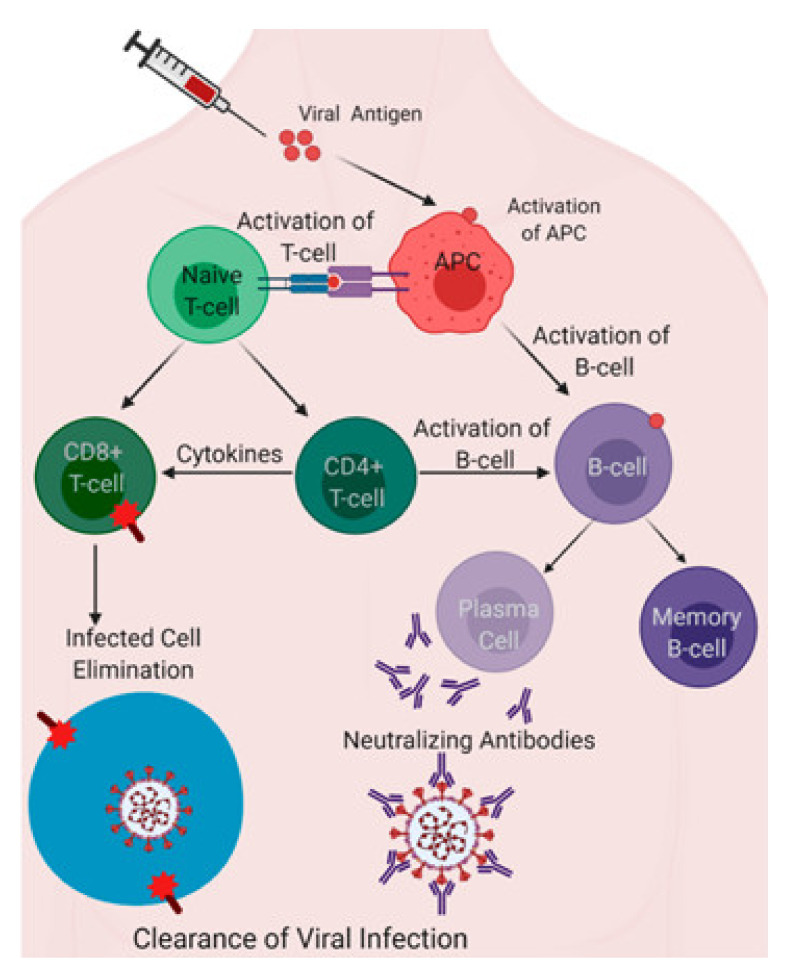
An antigen induces the activation of the antigen-presenting cells (APCs). The collaboration of the innate and adaptive immune arms can activate T-cell and B-cell immunity through fundamental immunological pathways, as represented to show how vaccine-elicited immune responses lead to the clearance of infection (modified from [168]).

The main aim of vaccination is to generate protective adaptive immunity in the form of antibodies and specific T-cell responses against SARS-CoV-2 with the involvement of innate immunity (Figure 4) [168].

The spike (S) proteins are molecules emerging from the surface of the virus, and their cleavage into two parts is an essential step for infection (Figure 3). One part (S1) of the S protein is a receptor-binding domain (RBD) that the virus uses to bind to the host receptor angiotensin-converting enzyme-2 (ACE2). The second subunit of the S protein (S2) mediates fusion with the cell membrane by forming a six-helical bundle via the two-heptad repeat domain to fuse with the cell membrane, enabling SARS-CoV-2 to infect the cell [6]. Antibodies specific to the S1 or S2 (see above, Section 2) proteins could neutralize and block the attachment and fusion of SARS-CoV-2 to the host cell. Therefore, neutralizing antibodies play an important role in viral clearance. The production of these antibodies specific to S1 or S2 could help to protect against future COVID-19 infection. Antibodies against the SARS-CoV-2 viral spike protein have been shown to have neutralizing effects. Current vaccines have been developed to elicit antibodies to the spike protein [6].

The production of antibodies against the S proteins is switched towards the IgM, IgG (mainly IgG1 and IgG3), and IgA classes. The levels of these antibodies peak 14 and 28 days after vaccination for IgM and IgG/IgA, respectively [168,169]. The serum concentrations of these antibodies can be measured in laboratories on a large scale to assess or follow up on vaccination and/or infections [169,170].

The CD4+ T-cell (Th1) response after vaccination can produce interferon-γ, tumor-necrosis factor-alpha, and interleukin (IL)-2. CD8+ T cells are also activated by the vaccines. Unfortunately, the measurement of T-cell responses is only possible in a limited number of laboratories. T-cell stimulation can be measured by INF-γ ELISpot, which directly measures TH1 activity [168].

If herd immunity among different nations is achieved at a desirable level in the postvaccination period, the COVID-19 pandemic could be stopped by the end of 2021 [171].

## 8. Conclusions

All the vaccines authorized for emergency use are safe and effective, with efficacy above 50% and up to 95%. No serious side effects from their administration have been reported, and they induce effective immune responses capable of protecting individuals against COVID-19 after the completion of the vaccination protocols. The appropriate choice of vaccine depends on different clinical, practical, and logistic parameters for countries and/or individuals. Important factors include the technology applied (modern or classical), the efficacy, vaccination protocol, the age group and health status of the vaccinee, storage requirements, side effects, and cost. More promising new vaccines are under development, and they are expected to add to the armory against SARS-CoV-2. Significant control of COVID-19 transmission by the end of 2021 is expected, through the achievement of widespread herd immunity in the postimmunization period.

## Figures and Tables

**Table 1:** Different leading vaccines currently authorized for use or are in advanced phase 3 clinical trials worldwide.

Vaccines	Types *
Pfizer–BioNTech	mRNA [10,11]
Moderna	mRNA [12]
CVnCoV (or CureVac)	mRNA [13]
Oxford–AstraZeneca	Vector-ChAdOx1 [14]
Sputnik V by Gamaleya	Vector-Ad5 and Ad26 [15]
Johnson and Johnson	Vector-Ad26 [16]
Ad5-nCoV (or Convidecia)	Vector-Ad5 [17]
Sinopharm	Inactivated [18]
Sinopharm Wuhan	Inactivated [19]
CoronaVac	Inactivated [20]
Covaxin (or BBV 152) by Bharat Biotech	Inactivated [21]
Novavax COVID-19	Protein subunit [22]
EpiVacCorona by Vector Institute	Synthetic protein [23]
ZF 2001	Protein–RBD dimer [24]

* Virus-like particle (VLP) vaccines are not approved for usage yet.

**Table 2:** Vaccines of different categories against SARS-CoV-2 virus in phase 1/2 clinical trials.

Vaccines	Types
ARCoV by China’s PLA Academy of Military Science (AMS), Suzhou Abogen Biosciences, and Walvax Biotechnology	mRNA [25]
Chulalongkorn University (Thailand)	mRNA [26]
LUNAR-COVID 19 by Arcturus Therapeutics and Duke-NUS Medical School	mRNA “self-amplifying” [27]
HGCO19 by Gennova Bio India and HDT Bio Seattle	mRNA “self-amplifying” [28]
VacEquity Global Health (Imperial College, U.K.)	Self amplifying RNA, skin implanted [29,30]
Covigenix VAX-001 by Entos Pharmaceuticals (Canada)	DNA, nucleocapsid gene [31]
DNA bacTRL-Spike by Symvivo (Canada)	DNA in a bacterial vector, oral [32]
CORVax 12	DNA for S protein and pIL-12 [33]
AG0302 by Japanese AnGes, Osaka University, and Takara Bio	DNA, skin injection [34]
Zydus Cadila (India)	DNA, skin patch [35]
INO-4800 by Inovio (Pennsylvania-based company)	DNA injection by skin device [36]
GeneOne Life Science (South Korea-based biotech company)	DNA encoding two proteins [37]
COVID-eVax	DNA fragment [38]
GRAd-COV2 by ReiThera (Italy) with Leukocare (Germany) and Univercells (Belgium)	Simian Ad GRAd vectored [39]
Vaxart (USA)	Ad5-vectored, oral [40]
AdCOVID by Altimmune Company	Ad5-vectored, nasal [41,42]
Convidecia (or Ad5-nCoV)	Ad5-vectored [43]
AdCLD-CoV19 by Cellid and IVI Biotech Co.	Ad5- and Ad35-vectored [44,45]
Flu-Covid Nasal by University of Hong Kong and Xiamen University	Influenza virus-vectored [46]
MVA-SARS-2-S by DZIF and IDT Biologika	MVA orthopoxvirus-vectored [47]
BriLife by Israel Institute for Biological Research	Vesicular stomatitis virus-vectored [48]
Recombinant vaccine by West China Hospital and Sichuan University	RBD of S protein in insect cells [49]
Adimmune (Taiwan-based manufacturer)	RBD of S protein [50]
Shionogi (Japanese pharmaceutical company)	Protein in insect cells [51]
Soberana 02 by Finlay Institute of Vaccines (Cuba)	RBD with tetanus toxoid [52]
CoVLP by Medicago and GSK (Canada)	Virus like-particles in plant cells [53]
Kentucky BioProcessing	Protein in plant cells (NBR) [54]
Dynavax by Clover Pharmaceuticals (China)	S-Trimer protein [55]
COVAXX (New York, USA)	Multitope peptide-based [56]
University of Tübingen (Germany)	Eight parts of two viral proteins [57]
COVAX 19 of Vaxine (Australia)	Protein subunit [58]
SpyBiotech and Serum Institute of India	Coronavirus RBD and HBsAg VLPs [59]
Mambisa by Center for Genetic Engineering and Biotechnology (Cuba)	RBD and HBV nasal spray [60]
Abdala by Center of Genetic Engineering and Biotechnology (Cuba)	RBD of S protein [61]
SK Bioscience (South Korea)	S protein [62]
Nanocovax by Nanogen Pharmaceuticals (Vietnam)	Protein-based [63,64]
COVAC by University of Saskatchewan (Canada)	Protein subunits [65]
CoviVac by Chumakov Centre (Russia)	Inactivated [66]
Valneva (France-based company)	Inactivated [67]
ERUCOV-VAC by Erciyes University (Turkey)	Inactivated [68]
QazCovid-in by RIBSP (Kazakhstan)	Inactivated [69]
COVIran Barekat by Shifa Pharmed (Iran)	Inactivated [70]
COVI-VAC (intranasal) by Codagenix	Live attenuated, nasal [71]

## Data Availability

Not applicable.
